# Identification of NUDT5 Inhibitors From Approved Drugs

**DOI:** 10.3389/fmolb.2020.00044

**Published:** 2020-03-31

**Authors:** Xin-Yu Tong, Xuan Liao, Min Gao, Bo-Min Lv, Xiao-Hui Chen, Xin-Yi Chu, Qing-Ye Zhang, Hong-Yu Zhang

**Affiliations:** Hubei Key Laboratory of Agricultural Bioinformatics, College of Informatics, Huazhong Agricultural University, Wuhan, China

**Keywords:** NUDT5, cancer, drug repositioning, molecular docking, molecular dynamic simulation

## Abstract

Recent studies have revealed the important role of NUDT5 in estrogen signaling and breast cancer, but research on the corresponding targeted therapy has just started. Drug repositioning strategy can effectively reduce the time and economic resources spent on drug discovery. To find novel inhibitors of NUDT5, we investigated the previously identified connectivity map-based drug association models and found eighteen FDA approved drugs as candidates. The molecular docking and molecular dynamic simulation were performed and revealed that fourteen organic drugs have the potential to bind the NUDT5 target. Eight representative drugs were selected to perform the cell line viability inhibition analysis, and the results showed that seven of them were able to suppress MCF7 breast cancer cells. Two drugs, nomifensine and isoconazole, showed lower IC_50_ than the known antiestrogens raloxifene and tamoxifen, and they deserve further pharmacodynamic investigations to test their feasibility for use as NUDT5 inhibitors.

## Introduction

The NUDIX hydrolases exist in all branches of life and catalyze the hydrolysis of a wide range of phosphate substrates (McLennan, 2006). So far, 24 NUDIX hydrolases genes have been identified in the human genome (McLennan, 2006). NUDT1 (also called MTH1) is one of the most investigated NUDIX hydrolases, and is found to be involved in certain diseases. NUDT1 can degrade a variety of substrates, including 8-OH-(d)GTP, 8-OH-(d)ATP, and 2-OH-(d)ATP. Because 8-OH-(d)GDP is usually produced by cellular oxidative stress events and causes genome damage, NUDT1 has been supposed to help maintain nucleic acid integrity and prevent oxidative-stress-related diseases, such as Parkinson’s disease and cancers (McLennan, 2006; Page et al., 2018). Another NUDIX hydrolases NUDT5 was also considered to be involved in cancer due to its 8-OH-(d)GDP hydrolysis activity. Knockdown of NUDT5 can suppress the proliferation of Hela cells and T47D cells but it didn’t induce DNA oxidative lesions (Li et al., 2017; Page et al., 2018). Other studies revealed that the 8-OH-(d)GDP degradation activity of NUDT5 depends on a basic condition (pH ≈ 10) instead of a physiological pH (Ito et al., 2011). These findings showed that NUDT5 has the potential to be a new anti-cancer target, but its role in cancer progression needs further investigation.

Recent studies revealed another function of NUDT5: driving nuclear ATP synthesis, which may play an important role in breast cancer. Previously, NUDT5 has been found to catalyze 5′diphosphoribose (ADP-ribose, ADPR) hydrolysis into ribose-5-phosphate (R5P) and adenosine 5′-monophosphate (AMP) (Wright et al., 2016; Page et al., 2018). Wright et al. (2016) proved that in the presence of pyrophosphate, NUDT5-catalyzed ADPR hydrolysis can generate both AMP and ATP. Nuclear ATP is the energy source for fundamental biological processes, such as chromatin remodeling and transcriptional change. Hormones like estrogen and progestin can induce such processes which could be carcinogenic (Le Dily et al., 2019). Wright et al. (2016) found that progestin or estrogen-induced nuclear ATP increasing, chromatin remodeling, and gene transcription changes are dependent on the activity of NUDT5 in breast cancer cell lines T47D and MCF7. NUDT5 was also found to be overexpressed in breast cancer patients and is correlated with a poorer prognosis and a higher risk of recurrence and metastasis (Pickup et al., 2019). These findings revealed the important role NUDT5 played in the estrogen signaling pathway and that it is thus involved in the pathogenesis of breast cancer. It also suggested that inhibitors of NUDT5 could be used as new drugs for the disease. Indeed, in the latest study, Page et al. (2018) found a series of targeted NUDT5 inhibitors and proved the inhibition of NUDT5 can block nuclear ATP synthesis, hormone signaling, and cell proliferation in breast cancer cells (Page et al., 2018). However, to our knowledge, Page et al.’s (2018) work is the only one aimed at finding targeted NUDT5 inhibitors; it is very urgent to develop other small-molecule drugs as therapies for corresponding diseases.

*De novo* drug discovery for a certain target is still an expensive and time-consuming task, while drug repositioning provides another solution. Connectivity map (cMap), which is a database comprising gene expression profiles for five types of human cell lines treated with 1309 agents, have been widely used in drug repurposing studies (Lamb et al., 2006; Iorio et al., 2010). Previously, 49 drug-induced transcriptional modules reflecting the association of drugs and gene expression were identified through analyzing cMap data with a biclustering method (Xiong et al., 2014), which is very helpful to clarify the pharmacological mechanisms and discover new activities of drugs (Li et al., 2014; Quan et al., 2015; Zhang et al., 2017). NUDT5 inhibitors have been proven capable of blocking estrogen signaling (Page et al., 2018), which is similar to the effect of antiestrogens. Therefore, in this study, drugs were first screened out from cMap agents who have similar biological effects to the known antiestrogens. Then, to evaluate whether the screened estrogen signaling inhibitors target NUDT5, the interactions between them and NUDT5 were analyzed by molecular docking and dynamics simulation. Finally, to evaluate the potential anticancer activities of the candidate NUDT5 inhibitors, some representative drugs were investigated in MCF7 cell line.

## Materials and Methods

### cMap Agents Biological Effects Similarity Analysis

The Tanimoto coefficient (*TC*) of each cMap agent pair was calculated to analyze the similarity of biological effects among cMap agents in our previous papers (Li et al., 2014; Quan et al., 2015). Briefly, 1309 cMap agents were connected with 49 gene modules in a non-orthogonal way. *TC* for each agent pair was calculated according to the following equation:

$$T\,C=\frac{N_{A\,B}}{N_{A}+N_{B}-N_{A\,B}}$$

where *N*_*A*_ and *N*_*B*_ are the numbers of modules connected with agents A and B, respectively, and *N*_*AB*_ is the number of modules connected with that A and B in common. A higher *TC* means agent pairs may have more similar biological effects (Li et al., 2014; Quan et al., 2015). In previous studies, we found that a *TC* > 0.45 of a pair of cMap agents represents a relatively reliable similarity in the bioeffect they cause (Li et al., 2014). In this study, 0.45 was set as the threshold to select potential estrogen signaling inhibitors. A previous study has revealed that the NUDT5 inhibitor can block the estrogen signaling pathway. cMap agents that have *TC* higher than 0.45 with antiestrogen tamoxifen, raloxifene, or fulvestrant were considered to be a candidate for estrogen signaling inhibitors. Then, based on the DrugBank database^1^, the approved drugs among them were identified.

### Molecular Docking

To verify and explore the possible interaction mechanism between the candidate drug and NUDT5 target, the molecular docking analysis was performed based on the known active site of NUDT5 by Molecular Operating Environment (MOE) (Vilar et al., 2008). The quality of each docking pose in the binding sites was assessed by using the root mean square deviation (RMSD) values and S score. The crystal structure of Homo sapiens NUDT5 (PDB code: 5nwh) was download from the Protein Data Bank^2^ and were prepared with the standard default procedure in the MOE. To validate the effectiveness of the docking method adopted in this study, the original ligand 7-5-(3,4-dichlorophenyl)-1,3,4-oxadiazol-2-yl]methyl]-1,3-dimethyl-8-piperazin-1-yl-purine-2,6-dione (TH5427) in the crystal structure of NUDT5 were extracted and redocked into the active site by MOE. The processed target and the original ligand were then analyzed for docking using the Triangular Matching docking method. S score and RMSD of 30 conformations in each docking configuration were generated for the interaction analysis.

### Molecular Dynamics

To further evaluate the binding energy between the NUDT5 target and candidate drug, molecular dynamics (MD) simulation and the MM/GBSA (molecular mechanics energies combined with Poisson–Boltzmann or generalized Born and surface area continuum solvation) calculation were performed based on the best binding mode of docking results by AMBER 14 software (Wang et al., 2019). The ff12SB force field were used for all amino acid residues. The system was neutralized by adding Na^+^ ions, and then the entire system was solvated into an 10 Å octahedral box of TIP3P. All ligand parameters were generated using the ANTECHAMBER module with BCC partial atomic charges in the AMBER 14. Subsequently, the prepared entire system was subjected to two steps of energy minimization. First, water molecules were minimized for 3,000 cycles with the complex structure constrained in 500 kcal mol^–1^ Å^–2^. Second, the entire system was minimized to a convergence of 0.001 kcal mol^–1^ Å^–2^ with 2,000 cycles of steepest descent and 1,000 cycles of conjugated gradient. The refined structure was then used for the binding energy calculation with MM/GBSA method, which has been applied to a large number of systems with varying success (Sun et al., 2014). The parameters of the other steps were set to default values.

### MCF7 Breast Cancer Cell Line Viability Inhibition Analysis

MCF7 cell line was purchased from Procell. All the tested drugs, medium, and other chemicals used in the cell culture were purchased from MedChemexpress. Cell Counting Kit-8 was purchased from Bimake. Microplate spectrophotometer (EON) was purchased from BioTek.

The MCF7 cells were cultured overnight in Dulbecco’s Modified Eagle’s Medium (DMEM), supplemented with 10% fetal bovine serum (FBS) in a humidified atmosphere of 5% CO_2_ and 95% air at 37°C. Then, the MCF7 cells were seeded in 96-well plates and incubated overnight. Next, MCF7 cells were incubated with different concentrations of tested drugs or solvent control for 48 h. All tested drugs were diluted with 1% DMSO. In the preliminary work, we investigated the appropriate dose and treatment regimen, which changed the duration and drug concentration of cells exposed to drugs. After pre-experiments, we identified five suitable concentration gradients for Nomifensine (at 84, 16.8, 3.36, 0.67, and 0.13 μM), Raloxifene (at 80, 16, 3.2, 0.64, and 0.13 μM), Mometasone (at 37.5, 18.75, 9.38, 4.69, and 2.34 μM), Bacampicillin (at 100, 20, 4, 0.8, and 0.16 μM), and four concentration gradients for Isoconazole (at 6.84, 1.37, 0.27, and 0.05 μM), Astemizole (at 15.63, 7.81, 3.91, and 1.95 μM), Flupentixol (at 80, 16, 3.2, and 0.64 μM), Tamoxifen (at 31.25, 15.63, 7.81, and 3.91 μM), and Fluvoxamine (at 400, 80, 16, and 3.2 μM) to calculate the IC_50_ of the drugs. For each experiment, three biological replicates were performed. After the drug treatment, the cell viability was measured using a Cell Counting Kit-8 following the manufacturer’s instructions and the absorbance at 450 nm for samples was measured using a microplate spectrophotometer. Finally, the half-maximal inhibitory concentration (IC_50_) value of each drug was calculated using Prism GraphPad Prism 7.0 software. The mean of triplicate experiments for each dose was used to calculate the IC_50_ values.

## Results and Discussion

### Screening of Potential Estrogen Signaling Inhibitors

cMap based drug repositioning can discover new activities of known drugs and is an effective way to reduce time and money resources spent on the drug discovery process. This strategy was used to find new NUDT5 inhibitors in this study. In our previous study, based on the cMap data, human genes were divided into 49 drug-induced transcriptional modules according to the expression changes induced by 1309 agents. A calculated module contains a set of genes whose expression is regulated (both up and down regulated) by a set of agents. The same modules were found to be enriched with agents that shared the same target pathways. So, the similarity in the connected modules between two agents can reflect the pharmacologic associations between them to a certain extent. Because NUDT5 plays an important role in the estrogen signaling pathway (Wright et al., 2016), and NUDT5 inhibitors can block the pathway (Page et al., 2018), one can expect that agents with NUDT5-inhibiting activity should connect similar modules with known antiestrogens.

Tamoxifen, raloxifene, and fulvestrant are widely used antiestrogens to treat estrogen receptor-positive breast cancers. These three drugs are also included in cMap agents. Eighteen FDA approved drugs of 1309 cMap agents were found to connect similar modules with at least one of the three antiestrogens. These drugs can be divided into six categories, including one inorganic salt (cobalt chloride), three sterol hormones, five antipsychotics, five antimicrobials, two antiallergics, and two antihypertensives. The structures of the 17 organic small molecule drugs are listed in Table 1. Among these drugs, sterol hormones genistein and quercetin have an anti-estrogen activity (Miodini et al., 1999), and finasteride has been reported to cause elevated estrogen levels (Ramot et al., 2009). The identification of these estrogen-signaling-associated drugs confirmed the effectiveness of our method. However, sterol hormones are more likely to affect hormone receptors rather than directly interact with NUDT5, and thus are excluded in the subsequent studies.

**TABLE 1:** The five categories of screened estrogen signaling inhibitors.

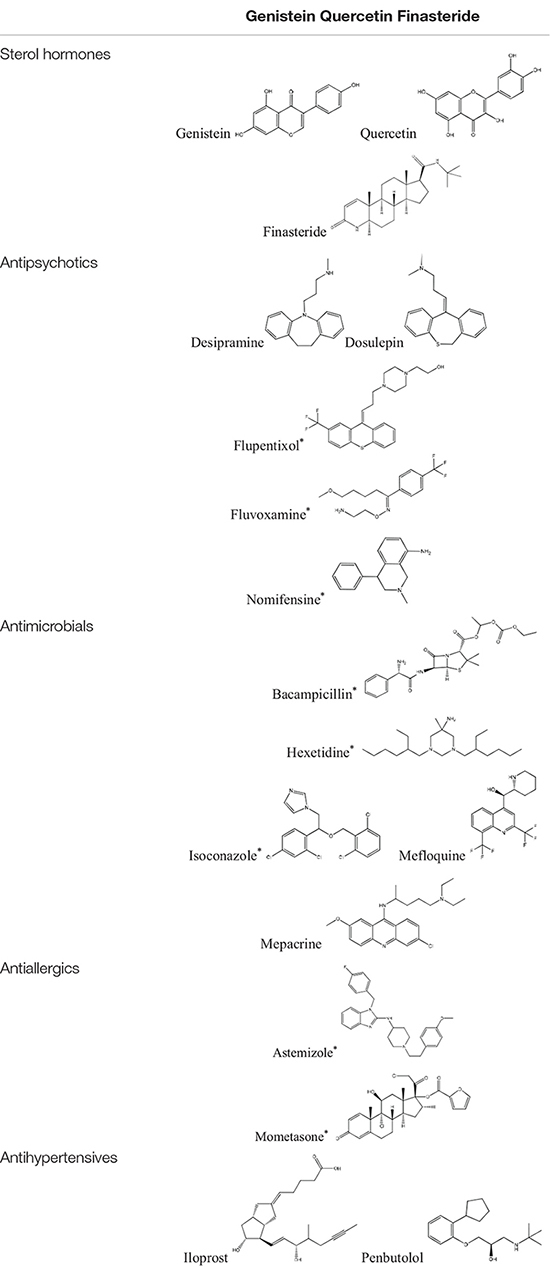

* Selected for the anticancer activity test.

### Molecular Docking and Molecular Dynamics

**FIGURE 1:**
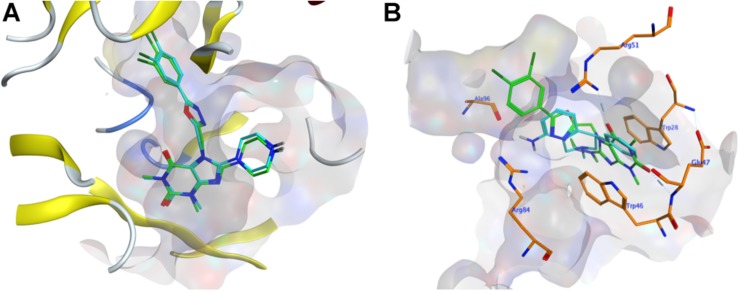
Molecular docking results. **(A)** The conformation comparison of TH5427 before and after the redocking. Orange-stick and cyan-stick represent original conformation and docked conformation, respectively, and the RMSD value was 0.338. **(B)** The conformation comparison between the docking conformation of nomifensine (cyan-stick) and TH5427 (green-stick) in the active site of the NUDT5 (PDB id: 5nwh), the pocket area was shown in light gray, the key residues were shown in orange-stick.

To evaluate whether the fourteen screened non-sterol hormonal potential organic estrogen signaling inhibitors can target NUDT5, molecular docking analysis was performed. Page et al. (2018) have crystallized NUDT5 in complex with its inhibitors. The structure of the complex (PDB code: 5nwh) revealed that the inhibitors occupy the substrate-binding cavity formed by the NUDT5 dimer and interaction with both subunits. According to Page et al’s. (2018) study, the receptor-ligand interactions between NUDT5 inhibitors and the targeted dimer is mainly made via the stacking interactions between amino acid residues Trp28 of chain A (TrpA28) and Trp46 of chain B (TrpB46), and hydrogen bonds to the basic side chain of Arg51 of chain A (ArgA51) and the acidic amide nitrogen of Glu47 of chain B (GluB47) (Page et al., 2018). So, these four residues were identified as the key interaction sites (Page et al., 2018). Before docking with the fourteen drug candidates, 7-[[5-(3,4-dichlorophenyl)-1,3,4-oxadiazol-2-yl]methyl]-1,3-dimethyl-8-piperazin-1-yl-purine-2,6-dione (TH5427), which is the original ligand of the crystal complex, performed redocking analysis to verify the rationality of the docking systems in MOE, and an RMSD value of 0.338 Å was obtained by comparing the conformation with its original binding mode in the active site (Figure 1A). In general, an RMSD value less than 2 Å indicated that the docking was rational for the entire system (Cole et al., 2005). Based on this verification, the fourteen organic small molecule drugs were docked with the NUDT5 dimer using the same MOE procedure. According to the S score of the respective docking, all fourteen drugs are comparable to the leading inhibitor TH5427 (Supplementary Figure S1 and Supplementary Table S1). Then, the interactions between the four key residues and the drugs were analyzed. The TrpA28, ArgA51, and TrpB46 of NUDT5 were close to the binding mode of all fourteen drugs, while GluB47 was close to eight of them (Supplementary Table S1). For example, as shown in Figure 1B, the binding mode of nomifensine (cyan-stick) was compared with the binding mode of TH5427 (green-stick), which illustrates that the nomifensine was capable of being a candidate drug not only because of the similar interactions with the key active sites of the NUDT5 dimer, but also because of additional residues including Ala96 and Arg84 of the pocket that are found to interact with nomifensine.

Next, we performed further MD simulations to predict the binding energy of these fourteen drugs using AMBER 14 software. The system calculates the final binding free energy (ΔGbind) from electrostatic energy (ΔEELE), van der Waals contribution (ΔEVDW), and electrostatic contribution to the solvation free energy(ΔEGB) using MM/GBSA. The ΔEELE and ΔEGB values show some disparities in the MD simulation of the fourteen drugs, while the ΔEVDW is consistent. The calculated final binding energy for all the drugs were negative values (Table 2) and showed they can form stable interactions with NUDT5 dimers. Although the ΔGbind shows some discrepancies with MOE docking results, the trends in the variation of energy are in agreement with the analyses from molecular docking. Taken together, the results of molecular docking and MD showed the fourteen organic estrogen signaling inhibitors have the potential to target NUDT5.

**TABLE 2:** Molecular dynamics results.

**Drugs**	**ΔEELE^a^**	**ΔEVDW^b^**	**ΔEGB^c^**	**ΔGbind^d^**
TH5427	–332.531	–56.175	362.0066	–33.9236
Desipramine	–11.3398	–37.3907	25.8853	–27.419
Dosulepin	–7.2606	–34.4205	14.8424	–31.3299
Flupentixol	–37.3965	–51.2613	38.2234	–57.0028
Fluvoxamine	–11.506	–43.6603	29.0184	–32.2934
Nomifensine	–1.0191	–34.6548	18.4698	–21.4164
Bacampicillin	–390.1033	–52.7505	404.2715	–45.6307
Hexetidine	–3.2582	–63.8221	21.8299	–52.6545
Isoconazole	–12.6222	–42.4328	24.6415	–35.5817
Mefloquine	2.3099	–45.8156	15.119	–34.2226
Mepacrine	–3.585	–53.7526	15.6189	–48.4387
Astemizole	–16.8307	–57.3159	42.4009	–39.1652
Mometasone	4.3643	–52.3134	22.9611	–23.3352
Iloprost	–48.9831	–49.7359	53.2914	–52.2866
Penbutolol	–13.1215	–47.3317	26.2063	–40.1834

*^*a*^the electrostatic energy calculated by MM force field. ^*b*^the van der Waals contribution from MM force field. ^*c*^the electrostatic contribution to the solvation free energy calculated by GB. ^*d*^the calculated final binding energy.*

### Potency of Candidate Compounds to Suppress Breast Cancer Cell Viability

Because our final goal is to find a new therapy for cancer, the anticancer activity of the selected drugs should be tested. To inspect the candidate compounds’ potency to suppress breast cancer cell viability, under the comprehensive consideration of drug categories and the results of molecular docking and MD, nine representatives from each category were chosen to perform the experimental validation. The antihypertensives were not tested because they are not available. The cytotoxic effects of the tested drugs on breast cancer MCF7 cells were measured by the half-maximal inhibitory concentration (IC_50_) and compared with the positive controls, which are approved estrogen receptor-positive breast cancer drugs raloxifene and tamoxifen and negative control DMSO solvent.

**FIGURE 2:**
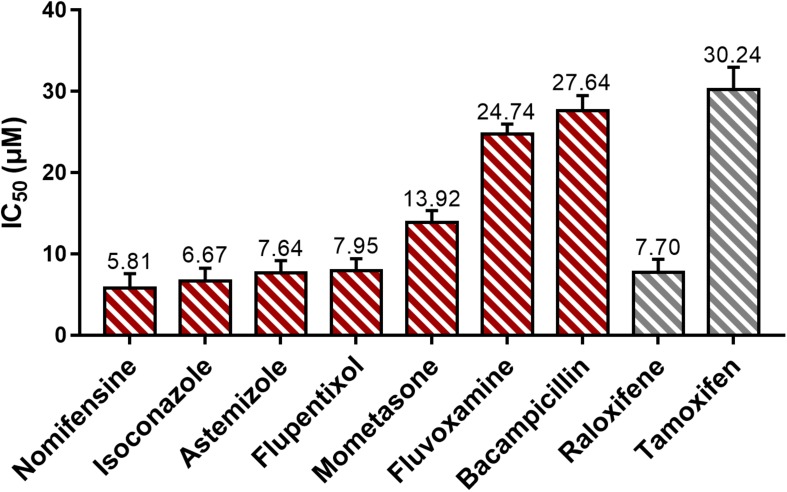
The cytotoxic effect of potential estrogen signaling inhibitors on MCF7 cell line. Red and gray represent tested drugs and positive controls, respectively. The IC_50_ values (the unit is μM) marked in the figure are the average of data from three independent experiments, bars represent the average standard deviations.

The IC_50_ for positive control tamoxifen is comparable to the previously measured values (31 μM) (Seeger et al., 2003) (Figure 2). Negative control did not exhibit observable inhibitory effects on treated cells. The tested drugs, except for hexetidine, have shown the observable cytotoxic effects on MCF7 cells and are dose-dependent. Among the active drugs, except for astemizole (Garcia-Quiroz et al., 2012), the remaining six drugs have not been reported to exhibit anticancer activity. The IC_50_ of nomifensine, isoconazole, and astemizole are lower than the two positive controls. The other four effective drugs also showed lower IC_50_ than tamoxifen (Figure 2). These results proved the anti-cancer activity of the seven drugs we detected is reliable.

Combined with the results of the molecular docking and MD, we found that the drugs – including nomifensine, isoconazole, and flupentixol – which directly interact with the key residues TrpA28 and TrpB46 in NUDT5 have a stronger ability to suppress breast cancer cell viability (Figure 2). In docking nomifensine to NUDT5, TrpB46 makes an arene-arene stacking interaction with the benzene ring of the drug (Figure 1B). For isoconazole and flupentixol, both TrpA28 and TrpB46 form arene-arene stacking interactions with their benzene rings (Supplementary Figure 1A,B). The direct interaction with these key residues were lacking in the other four drugs (Supplementary Figure 1C,F). This phenomenon implies the critical role of the residues played in NUDT5 dimer, and these drugs may act through targeting NUDT5. An exception is astemizole, which has no direct interaction with the key residues but has a low IC_50_ value. A possible explanation is that astemizole can suppress cancer cells in other ways. Indeed, astemizole has been reported to be able to synergize the antiproliferative effect of calcitriol through downregulating CYP24A1 and upregulating VDR (Garcia-Quiroz et al., 2012). Taken together, these findings suggest that the seven drugs with anti-cancer activity may target NUDT5, and three of them may target the key functional amino residues.

## Conclusion

NUDT5 plays important roles in the estrogen signaling pathway, and thus could be involved in the pathogenesis of breast cancer. The inhibitors of NUDT5 could represent new therapies for the disease. In this study, 18 approved drugs were firstly selected from 1309 cMap agents according to the similarity to the known antiestrogens in their biological effects. The molecular docking and molecular dynamic analyses revealed that the fourteen non-sterol-hormones organic drugs have the potential to bind NUDT5. Seven of eight of these drugs have shown the ability to suppress the viability of the MCF7 breast cancer cell line. Nomifensine and isoconazole, which may interact with the key functional residues in NUDT5, have lower IC_50__t_han than known antiestrogens raloxifene and tamoxifen. The above results show the application value of the drug repositioning tactic in finding novel NUDT5 inhibitors. The selected drugs deserve further experimental investigations to verify whether they can bind NUDT5 and inhibit its activity.

## Data Availability Statement

Publicly available datasets were analyzed in this study. This data can be found here: http://www.broadinstitute.org/cmap/.

## Author Contributions

Q-YZ, X-YC, and H-YZ conceived and designed the study. MG performed the cMap agents biological effects similarity related analysis. X-YT and B-ML performed the cell experiments. XL and X-HC performed the molecular docking and molecular dynamic analyses. X-YT, XL, and X-YC wrote the manuscript. Q-YZ and H-YZ revised the manuscript.

## Conflict of Interest

The authors declare that the research was conducted in the absence of any commercial or financial relationships that could be construed as a potential conflict of interest.

## Footnotes

1 https://www.drugbank.ca/

2 https://www.rcsb.org/
