# Supplementary material for: Identification of NUDT5 Inhibitors From Approved Drugs
Source: Front Mol Biosci. 2020 Mar 31;7:44. doi: 10.3389/fmolb.2020.00044 (PMC7145388; doi:10.3389/fmolb.2020.00044)
Supplement: Supplementary file 1 [file Data_sheet_1.pdf]

Supplementary Material

1 Supplementary Figure and Table

1.1 Supplementary Figure

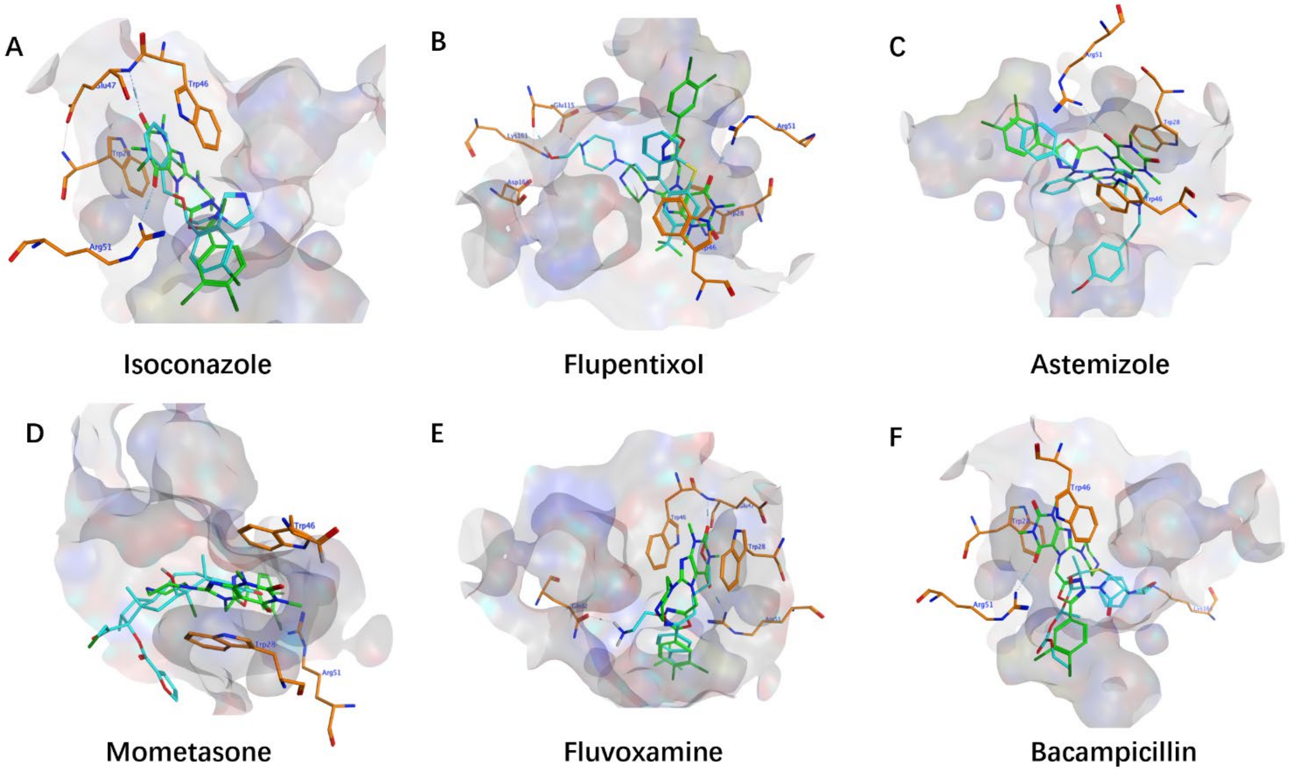

Supplementary Figure 1. The interactions among drugs (cyan-stick) and TH5427 (green-stick) in the active site of the NUDT5 (PDB id: 5nwh), the pocket area was shown in light grey, the key residues were shown in orange-stick.

1.2 Supplementary Table

Supplementary Table 1 Molecular docking results

Drugs	Key binding sites	S	rmsd	rmsd_refine	E_conf	E_place	E_score1	E_refine
TH5427	TrpA28, ArgA51, TrpB46,GluB47	-9.28812	0.33821	4.099664	-311.516	-86.2393	-16.8203	-81.6036
Desipramine	TrpA28, ArgA51, TrpB46	-6.3497	2.1009	-12.4099	-49.8210	-10.0540	-25.4108	-6.3497
Dosulepin	TrpA28, ArgA51, TrpB46,GluB47	-6.1419	3.5809	75.4757	-39.6498	-9.4430	-19.5538	-6.1419
Flupentixol	TrpA28, ArgA51, TrpB46,GluB47	-7.5712	1.7306	184.1463	-46.4759	-11.4540	-48.2583	-7.5712
Fluvoxamine	TrpA28, ArgA51, TrpB46,GluB47	-7.4620	1.1325	84.8473	-85.7743	-10.9010	-39.4306	-7.4620
Nomifensine	TrpA28, ArgA51, TrpB46,GluB47	-5.9876	1.6543	31.5381	-62.7463	-10.0555	-27.6790	-5.9876
Bacampicillin	TrpA28, ArgA51, TrpB46	-8.2109	1.5211	82.5701	-106.6023	-11.1237	-45.6010	-8.2109
Hexetidine	TrpA28, ArgA51, TrpB46,GluB47	-7.4953	2.0327	85.9626	-66.4767	-10.8280	-23.6863	-7.4953
Isoconazole	TrpA28, ArgA51, TrpB46,GluB47	-7.3561	1.2545	-7.0514	-74.3754	-11.0271	-35.2012	-7.3561
Mefloquine	TrpA28, ArgA51, TrpB46,GluB47	-7.4198	1.2248	111.5678	-66.6570	-11.8203	-33.0587	-7.4198
Mepacrine	TrpA28, ArgA51, TrpB46,GluB47	-7.9458	1.3889	-30.2148	-82.2922	-11.6019	-39.3995	-7.9458
Astemizole	TrpA28, ArgA51, TrpB46	-8.3492	2.4369	-33.6297	-61.2248	-11.4556	-32.9665	-8.3492
Mometasone	TrpA28, ArgA51, TrpB46	-5.8447	1.8692	183.9413	-51.6825	-8.8172	-21.5552	-5.8447
Iloprost	TrpA28, ArgA51, TrpB46	-8.2081	2.2149	23.8427	-81.8911	-11.5352	-43.4889	-8.2081
Penbutolol	TrpA28, ArgA51, TrpB46	-6.5346	3.0313	-31.0387	-82.0900	-12.5252	-28.8742	-6.5346
